# The Arc of Riolan artery may serve as the only pathway for lymphatic metastasis in advanced splenic flexure cancer

**DOI:** 10.1007/s10151-025-03275-4

**Published:** 2026-01-21

**Authors:** J. H. Tan, A. M. Zuki, S. F. Chiew, S. H. Kim

**Affiliations:** 1 Colorectal Unit, Department of Surgery, Faculty of Medicine, Universiti Malaya Medical Centre, 59100 Kuala Lumpur, Malaysia; 2 Department of Pathology, Universiti Malaya Medical Centre, Kuala Lumpur, Malaysia; 3 Monash Clinical School Johor Bahru, Monash University, Johor Bahru, Malaysia

**Keywords:** Arc of Riolan (AoR), Accessory middle colic artery (aMCA), Central vascular ligation (CVL), Colorectal cancer

## Abstract

**Background:**

Colon cancer located at the splenic flexure exhibits dual lymphatic drainage via the left middle colic artery (lt-MCA) to the superior mesenteric artery (SMA) system and the left colic artery (LCA) to the inferior mesenteric artery (IMA) system. However, an additional pathway—the Arc of Riolan (AoR) artery, central anastomotic vessels connecting the SMA and IMA—may also serve as a route for metastasis. This case highlights the importance of central vascular ligation of the AoR in splenic flexure cancer.

**Case:**

We present a rare case of isolated AoR lymph node metastasis in a 72-year-old male with advanced splenic flexure cancer. The patient presented with multiple synchronous tumors (splenic flexure, sigmoid, and rectum) and underwent extended left hemicolectomy with central vascular ligation (CVL) of the AoR, revealing metastatic involvement exclusively in AoR nodes. This represents the first documented case of isolated AoR nodal metastasis, emphasizing the need for AoR lymphadenectomy when present.

**Discussion:**

Recent studies suggest that accessory middle colic arteries (aMCA) and AoR may represent the same anatomical structure, with metastasis rates of 3.7–6.3% in corresponding nodes. Our findings support that AoR should be considered a critical target for CVL in splenic flexure cancer, particularly when identified pre- or intraoperatively.

**Conclusions:**

Surgeons should recognize AoR as a possible isolated metastatic pathway and perform thorough nodal dissection along this vessel when present to ensure optimal oncologic outcomes.

**Supplementary Information:**

The online version contains supplementary material available at 10.1007/s10151-025-03275-4.

## Introduction

It is well known that colon cancer located in the splenic flexure has a dual pathway of lymph node metastasis—one through the left branch of the middle colic artery (lt-MCA) to the superior mesenteric artery (SMA) system and the other through the left colic artery (LCA) to the inferior mesenteric artery (IMA) system. An oncologic resection is indicated on the basis of this concept [1].

The Arc of Riolan (AoR) is an additional artery directly connecting the IMA system to the SMA system, found in less than 20% of individuals, to supply the descending/splenic flexure colon [2] [3]. The current report describes a rare case of isolated AoR lymph node metastasis from an advanced splenic flexure cancer, skipping pericolic node involvement. This case highlights the importance of central vascular ligation of AoR when this artery is present in splenic flexure cancer.

## Case report

A 72 year-old male complained of 3 months of intermittent left-sided abdominal pain, altered bowel habit, and loss of appetite. He was frail with Eastern Cooperative Oncology Group function 1. A hard mass was detected at 6 cm from the anal verge on digital rectal examination.

He underwent a colonoscopy assessment that revealed four differently located tumors. Three of them were circumferential fungating masses in the rectum at 6 cm from the anal verge, in the sigmoid colon, and in the splenic flexure, respectively. The biopsy showed adenocarcinoma. One 1.5 cm polyp was also detected in the ascending colon, which showed dysplasia on biopsy, and was unable to be excised endoscopically owing to difficulty reaching it with a redundant colon.

**Fig. 1:**
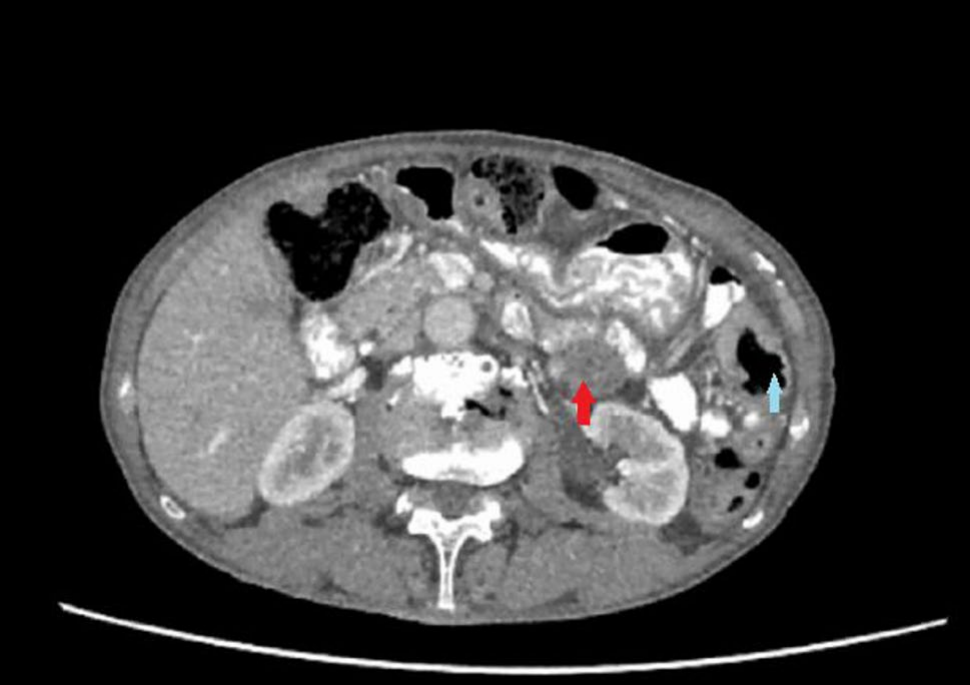
Computed tomographic scan shows an enlarged lymph node in the central mesentery (red arrow) with a splenic flexure tumor (blue arrow)

Computed tomography (CT) scans of the thorax, abdomen, and pelvis showed no distant metastasis and revealed resectable tumors in the splenic flexure (cT3N1M0, 3.5 cm in length), sigmoid colon (cT3N0M0, 5 cm), and rectum (cT4aN0M0, 4.5 cm). There were two enlarged central mesenteric lymph nodes around the splenic flexure cancer (Fig. 1). Magnetic resonance imaging of the pelvis revealed a midrectal tumor possibly involving the anterior peritoneal reflection with no regional pelvic nodes (mrT4aN0).

**Fig. 2:**
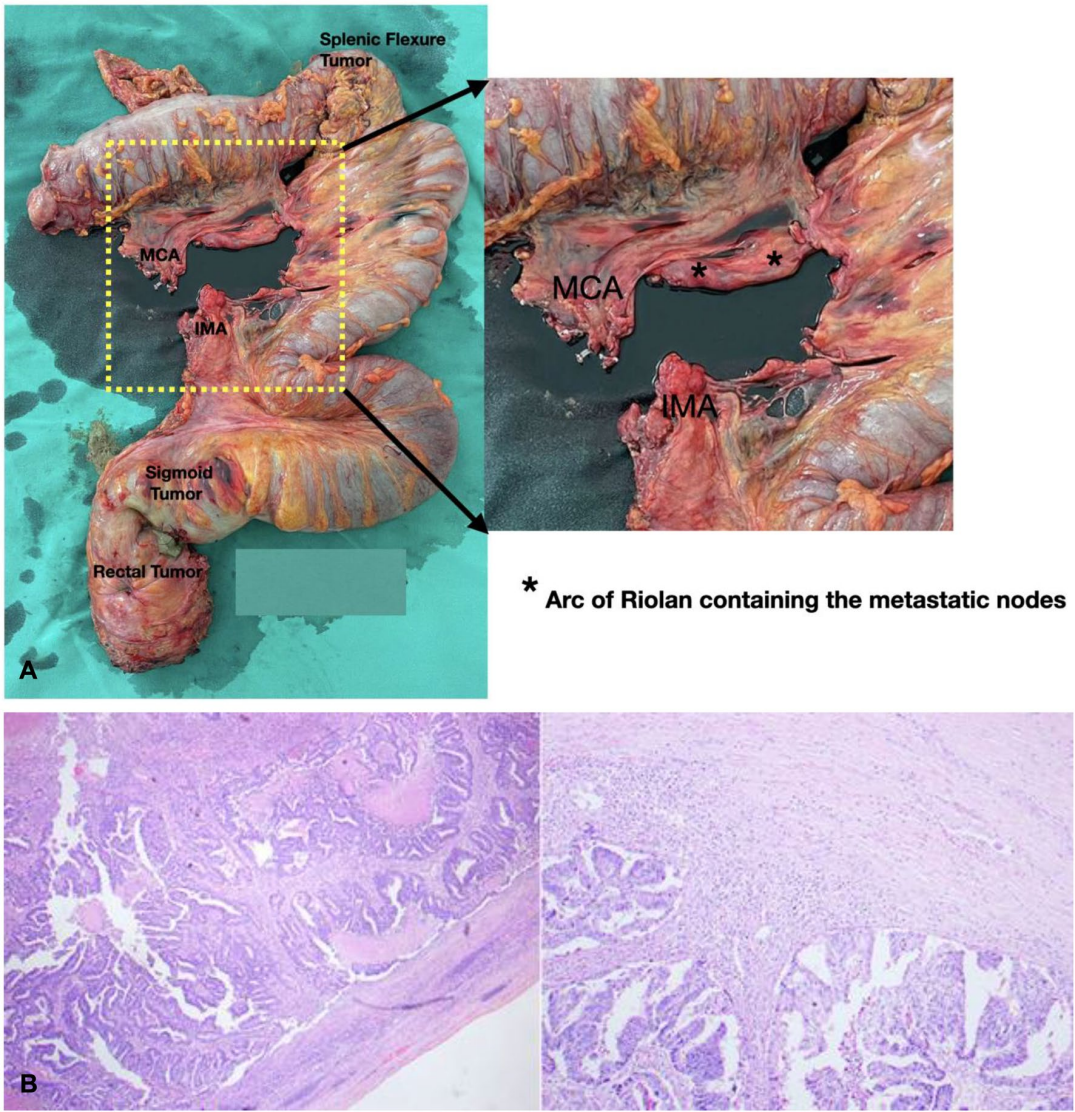
**A** The resected specimen obtained from Deloyers procedure. Central vascular ligation of the inferior mesenteric artery and the middle colic artery, along with central lymph node dissection is clearly seen. The resected Arc of Riolan (AoR) lymph nodes are also shown (*). **B** Histology of the AoR lymph node (left) and the splenic flexure tumor (right) at 4× magnification showing infiltrative malignant cells in complex cribriforming architectures with luminal dirty necrosis, moderate nuclear pleomorphism, and brisk mitosis

Upon the tumor board decision, short-course radiotherapy was administered to the rectal tumor. Then, low anterior resection and extended left hemicolectomy with retrograde right colon–rectum anastomosis (Deloyers procedure) were performed laparoscopically in an en-bloc specimen, together with a local excision of the ascending colon polyp via a separate colotomy (Supplementary Video 1). Postoperatively, oral feeding was slowly advanced owing to small bowel ileus; then, the patient was discharged after 8 days. Final histopathology revealed that all lesions were mucinous adenocarcinomas with clear margins. The lesions at the splenic flexure, sigmoid colon, and rectum were classified as pT3, while the ascending colon polyp was pT2. A total of 50 lymph nodes were examined, with 2 positive nodes found only at the AoR (Fig. 2). One epicolic lymph node separately excised from the ascending colon during the local excision of the polypoid tumor was also negative. Considering the staging of the splenic flexure cancer and a potential risk of nonoptimal resection of the pT2 ascending colon cancer, adjuvant chemotherapy (5-fluorouracil and oxaliplatin for eight cycles) was given, and the patient remained disease free at 18 months follow-up.

## Discussion

The common lymphatic drainage pathways for splenic flexure tumors are via either LCA or lt-MCA [4]. Classically, nodal dissection for splenic flexure tumors has focused more frequently on LCA [4]. A retrospective study from Korea reviewed 47 patients over a 14-year cohort who underwent laparoscopic D3 resection for splenic flexure tumors. The authors described the extent of nodal dissection, emphasizing the left colic artery but extending more to the roots of the MCA and IMA in advanced-stage cases [1]. Notably, aberrant or accessory arteries sometimes supply the splenic flexure and may serve as pathways for lymphatic spread in splenic flexure cancer [3]; However, central nodal dissection along these accessory vessels has rarely been reported.

In 2023, the first lymph node metastasis mapping study for splenic flexure colon cancer was published by researchers in Japan [5], further emphasizing the unpredictable nature of lymphatic drainage in this region [5]. The authors described the presence of the accessory middle colic artery (aMCA) was observed in 41.8% (64/153) of cases. Prior studies have documented the presence of an additional or aberrant artery in the left side of the colon at highly variable rates, ranging from 4% to 49.2% [3, 6]. This variability may be attributed to differing definitions of the same vessels in this region, classified either as aMCA [5], AoR [3], or as a left accessory aberrant colic artery [6]. The AoR may be distinctly described differently from the aMCA in Japanese literature, as aMCA is defined as additional artery, separate from the usual middle colic artery, that branches mainly from the central side of SMA and supplies the splenic flexure [5, 7].

However, on the basis of the recent study by Kuzu et al. of 107 anatomical dissection series [8], the authors described AoR as central anastomotic vessels (arterial anastomoses between the SMA and the IMA that are adjacent to the inferior mesenteric vein (IMV) at the level of the duodenojejunal junction and at the lower border of the pancreas, which is seen in 17 patients (16%). The aMCA vessel can form part of the central anastomotic vessels (10 out of 17) or, at times, it forms part of the intermediate anastomotic vessels (4 out of 49). Therefore, in the majority of cases, the aMCA is part of the AoR or vice versa [8, 9]. In the current authors’ opinion, these two arteries are the same in most cases.

Watanabe et al. noted that when there was an aMCA identified, metastasis rates at the aMCA were 6.3% at intermediate nodes and 3.7% at apical nodes [5]. These findings suggest that when an aMCA is present, it should be the target for central vascular ligation (CVL). The authors also noted that when metastasis occurs in the aMCA nodes, the lymph nodes around the apical region of the LCA and lt-MCA are negative; however, metastases are still present in the intermediate nodes of the LCA and lt-MCA in 10–20% of cases. Though this may suggest CVL of the LCA and lt-MCA at the apical region may not be necessary owing to low oncologic yield, we still routinely recommend CVL of the lt-MCA and LCA in all splenic flexure cancers owing to its conventional advantages in most splenic flexure cancers. Even when the aMCA is absent, the metastasis rates are 2.3% for the lt-MCA apical nodes and 1.4% for the LCA apical nodes [5]. Thus, in the absence of an aMCA, we believe that central lymph node dissection along the lt-MCA and LCA should be the primary oncologic dissection.

**Fig. 3:**
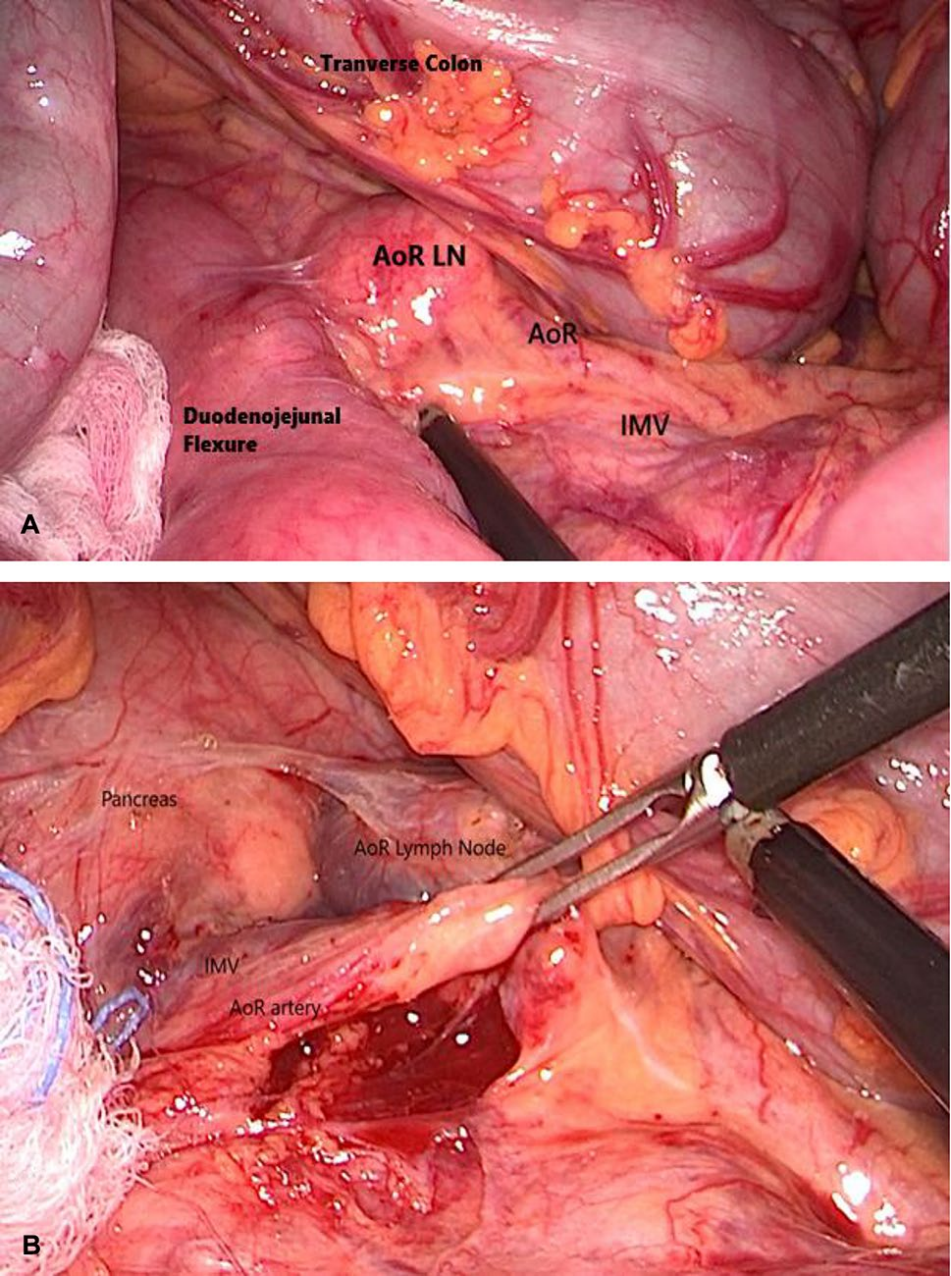
**A** Under the upward traction on the transverse colon, the enlarged Arc of Riolan (AoR) node is visualized along the AoR artery next to the duodenojejunal flexure. **B** Once the duodenojejunal flexure is dissected off, a laparoscopic clamp is holding the AoR and the inferior mesenteric vein (IMV) to perform a separate AoR lymphadenectomy. The AoR anteriorly crossing the root of the IMV close to the inferior border of the pancreas is clearly visualized

In our case, the enlarged central mesenteric lymph nodes identified on the preoperative CT scan were intraoperatively found to be located along the AoR (Fig. 3). A high ligation of the IMV, along with a ligation of the AoR, allowed for an optimal oncologic resection with complete nodal dissection. Actually, the AoR is a part of the mesocolon, then it should be included during a standard left hemicolectomy, regardless of a D2 or D3 dissection. This approach, however, may not be standardized in clinical practice. The presence of the AoR indicates the need for careful mesocolic dissection around the AoR and IMV, as demonstrated in this case (Fig. 3, Supplementary Video 2).

Our literature review identified two published cases with technical videos demonstrating aMCA central lymph node dissection [10, 11], both of which were negative for nodal metastasis. In contrast, our case revealed pathological involvement of the AoR lymph node—a notable finding given that the AoR serves as the exclusive lymphatic drainage route in this region. To our knowledge, this represents the first reported case of the isolated AoR lymph node metastasis of the splenic flexure cancer in literature. This case may complicate the interpretation of isolated nodal spread, whether originating from the splenic tumor alone or not, given the presence of multiple colonic and rectal tumors. However, the splenic flexure tumor was the closest to the pathological AoR node, and this node could not be considered a regional node for the sigmoid colon or the rectum.

Furthermore, this case may suggest that the presence of the AoR can be a plausible explanation for the occurrence of aberrant nodal metastasis from left-sided colon cancer to the territory supplied by the SMA system or, conversely, from right-sided colon cancer to the territory supplied by the IMA system [12].

## Conclusions

In splenic flexure colon cancer, lymphatic spread via the AoR artery may occur as an isolated pathway. Therefore, nodal dissection along the AoR is a critical step in CVL, when this artery is present.

## Supplementary Information

Below is the link to the electronic supplementary material.

Supplementary file1 Supplementary Video 1 (to depict the case summary and the whole procedure performed) (MP4 97841 KB)

Supplementary file2 (MP4 60737 KB)

Supplementary file3 Supplementary Video 2 (to improve understanding of AoR artery ligation for AoR lymphadenectomy) (MP4 131849 KB)

Supplementary file4 (DOCX 7815 KB)

## Data Availability

No datasets were generated or analyzed during the current study.
