# Supplementary material for: The Arc of Riolan artery may serve as the only pathway for lymphatic metastasis in advanced splenic flexure cancer
Source: Tech Coloproctol. 2026 Jan 21;30(1):30. doi: 10.1007/s10151-025-03275-4 (PMC12858470; doi:10.1007/s10151-025-03275-4)
Supplement: Supplementary file 4 — Supplementary file4 (DOCX 7815 KB) [file 10151_2025_3275_MOESM4_ESM.docx]

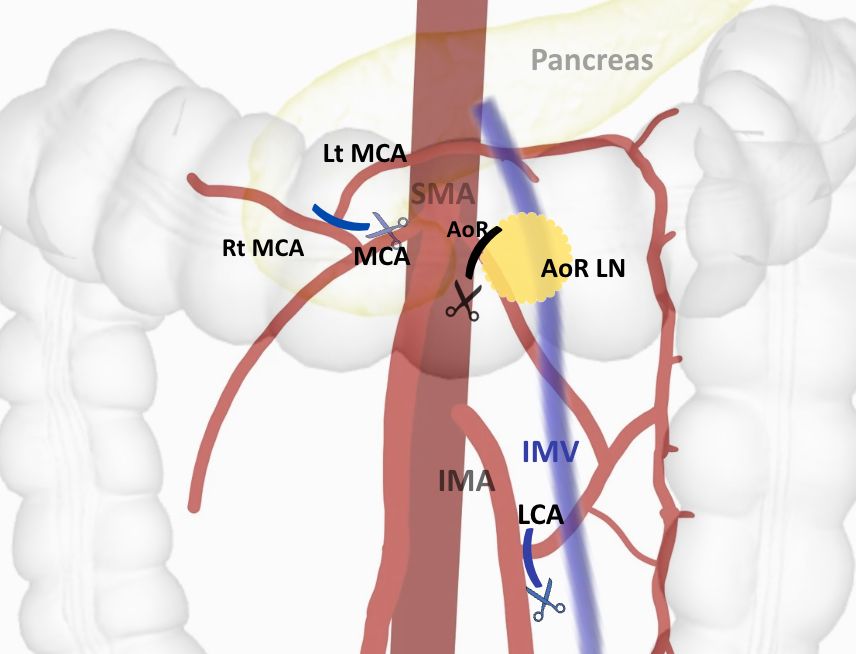


***Supplementary Figure 1:*** *Vascular anatomy in related to central vascular ligation (CVL) for a splenic flexure cancer. The blue-marked vascular ligation refers to a standard CVL for a splenic flexure cancer and the black-marked vascular ligation refers to an additional CVL when AoR is existing. In this patient, IMA and MCA were ligated at the root due to synchronous sigmoid and rectal cancers and a part of performing Deloyers procedure. [SMA (superior mesenteric Artery); IMA (inferior mesenteric artery); IMV (inferior mesenteric vein); MCA (middle colic artery); Rt MCA (right branch of middle colic artery); Lt MCA (left branch of middle colic artery); LCA (left colic artery); Arc of Riolan (AoR); AoR LN (Arc of Riolan lymph node)].*
